# A histological Study of the Latent Period of Hepatoma Grafts

**DOI:** 10.1038/bjc.1961.71

**Published:** 1961-09

**Authors:** Mary A. Head, Helen M. Laird

## Abstract

**Images:**


### A HISTOLOGICAL STUDY OF THE LATENT PERIOD OF HEPATOMA GRAFTS

MARY A. HEAD AND HELEN M. LAIRD*

*From the Cancer Research Department, Royal Beatson Memorial Hospital, Glasgow*

Received for publication July 29, 1961

IN their study of transplanted hepatomas in inbred mice Andervont and Dunn (1952) noted a long latent period before grafts became palpable and that the majority of their grafted tumours grew in only a proportion of recipients in the same strain of inbred mice. They considered that non-genetic factors were operating against successful establishment of transplantable primary hepatomas. They also pointed out that the long latent period might account for failure of some of the grafts to take before the experiment was ended and that studies during this period of slowly developing tumours might be helpful in solving the problem, in clinical medicine, of how tumour cells survive in cases where recurrences or metastases occur many years after removal of a primary tumour.

Our experiments have been carried out to study the histological appearances of slowly growing hepatoma grafts each week during the latent period, which was taken as the time between grafting and the detection of a palpable tumour by weekly examination. The tumour studied is a spontaneous hepatoma from a 23 months old RIIIf male breeder and was originally grafted by Dr. B. D. Pullinger, in this Department. A large, irregular slightly nodular swelling was found in the left posterior lobe of the liver. It was paler in patches than the rest of the liver which appeared normal. There were some very large vessels on the surface of the tumour making grooves in it. On histological examination the tumour is seen to be a trabecular hepatoma. It is composed of cords of liver-like cells in which the cytoplasm is abundant and the nucleus has the size, appearance and prominent nucleoli of normal liver cells. Mitoses are fairly frequent (66 per 100 high power fields). Some unusual intracytoplasmic inclusion bodies are present (Laird, 1955 ; Laird and Head, 1956).

The hepatoma was grafted subcutaneously into 10 male RIIIf mice for the first passage. Half of the grafts grew after a latent period of 5 to 14 months before they became palpable. In the remaining 5 mice, discrete groups of apparently viable hepatoma cells were found in sections of the site of injection after 8 to 12 months. The tumour was therefore considered a suitable one in which to study the histological appearances during the latent period. In the second generation, female as well as male mice were grafted. One of 2 males and none of 5 females killed after one year had successful grafts. In the third generation 6 of 7 male mice had palpable grafts. In the fourth generation 6 of 8 males and none of 13 females had successful grafts (Head and Laird, 1957). The higher incidence of primary spontaneous hepatomas in male than in female mice has previously been

* Working under a full-time grant from the British Empire Cancer Campaign.

noted by various authors (Burns and Shenken, 1940 ; Andervont, 1950 ; Agnew and Gardner, 1952).

The 5th, 6th and 8th transplant generations have been studied in the present experiment (Head and Laird, 1958 and 1959). The 7th generation transplants were used in another experiment.

The appearances of the grafted tumour are very similar to those of the original spontaneous primary hepatoma. Parts of it are very liver-like, both on naked eye and histological examination. The larger grafts are usually pale brown and often have cystic, haemorrhagic or necrotic areas. Microscopically they are composed of cords of cells in which the cytoplasm is abundant and the nucleus has the size and appearance, with prominent nucleoli and well defined nuclear membrane, of normal liver parenchymal cells. Although mitoses are not very frequent and cells are well differentiated they are considered to be malignant tumours, as metastases have been found in the lungs of mice in previous experiments with this same type of slowly growing hepatoma. The vascular supply to the grafts is usually very good and enlarged vessels are seen around them.

### MATERIAL AND METHODS

Four groups of mice were grafted :

1. 51 male mice RIIIf and ($C_3Hf \times$ RIIIf) $\times$ RIIIf $F_1$ with a 5th generation transplant of the grafted tumour.
2. 21 female and 5 male mice ($C_3Hf \times$ RIIIf) $\times$ RIIIf $F_1$ with a 6th generation transplant.
3. 14 female and 5 male mice RIIIf and ($C_3Hf \times$ RIIIf) $\times$ RIIIf $F_1$ with a 6th generation transplant.
4. 24 male mice RIIIf and ($C_3Hf \times$ RIIIf) $\times$ RIIIf $F_1$ with an 8th generation transplant.

*First Series* : 51 *male mice*

25 RIIIf and 26 ($C_3Hf \times$ RIIIf) $\times$ RIIIf $F_1$ mice aged 3 months were used They were anaesthetised with Bromethol, a small mid-line incision was made and a portion of graft inserted into a pocket dissected in the subcutaneous tissue of the right flank with a pair of blunt pointed forceps. The tumours to be grafted were removed aseptically from the donor and placed in a sterile Petri dish. Portions of the tumour were chosen which were non-necrotic, non-fatty and non-haemorrhagic. The tissue was kept from becoming dry by keeping the lid of the dish moist with sterile saline. Approximately equal portions of tissue were grafted. The mice were numbered by ear-marking and one pure line and one back cross were chosen at random to be killed each week, after grafting. Three mice were killed in the 1st week, and three in the 15th week (in each case the third mouse was ill), two additional mice were killed at the 11th week, as the first two had palpable tumours. A third mouse died at the 16th week and only one was killed at the 21st week in order to leave five mice alive till they died or had large tumours. This was done in order to compare the number of palpable tumours at the later stage with the various appearances of the graft sites in the earlier stage of the experiment, to obtain further information about the length of the latent period and to find whether or not metastases occurred from the large slowly growing grafts.

The site of grafting could be made out at autopsy, when the skin was reflected, either macroscopically or by using a dissecting microscope with $\times 7 \times 10$ magnification. It appeared either as tiny white nodules or as a small pigmented area. The graft and surrounding tissue were pinned out on cork, fixed in Bouin's fluid (sometimes stained in bulk) and then prepared for histological examination. Spiral sections were stained with haematoxylin and eosin or haemotoxylin eosin heloxine tartrazine. The white nodules consisted of small encapsulated groups of hepatoma cells. Sections were stained to try to identify the pigment in some of the hepatoma cells and in the surrounding tissues.

Groups of hepatoma cells were found in one or more of the mice examined each week until the 18th, with the exception of the 7th and 9th weeks when no graft could be found. In mice killed from the 19th to the 21st week no growing hepatoma cells were seen. There was no apparent difference in the grafts in the RIIIf and the back cross mice so they will be described together.

*First week.*—In one mouse which was killed because it was ill, vacuolated hepatoma cells lying singly and in small groups were found in a small fibrous nodule about 1 mm. diameter (Fig. 1). In two other mice killed after 1 week, although the site of the graft could be seen macroscopically as a small yellow nodule, no liver cells were found microscopically.

*Second week.*—In one mouse the graft site was seen through the dissecting microscope as an area of tiny white nodules. Histologically these were seen to be small groups of hepatoma cells which showed less vacuolation than those in in the first week and were almost encapsulated. In the other mouse the graft site was hardly visible and no liver cells were found.

*Third week.*—The site of grafting in both mice was less well defined than in the 1st and 2nd weeks. A group of well preserved hepatoma cells was found in one mouse and in the other a few nucleated cells which were possibly hepatoma cells were seen beside an area of reaction tissue.

*Fourth week.*—In one mouse three small white areas were seen through the dissecting microscope after 10 minutes' fixation in Bouin's fluid. Histologically 4 groups of well preserved hepatoma cells were found. The nuclei had prominent nucleoli and the cytoplasm was densest nearest the nuclei. The groups of cells were surrounded by fat (Fig. 2). In the other mouse a small brown graft was found macroscopically. Large masses of pigment were found histologically in cells of uncertain nature other than the hepatoma cells which formed a small group. There was no clear cut margin between the two types of cells and a few hepatoma cells were scattered in the surrounding tissue.

*Fifth week.*—Macroscopically a tiny white nodule and pigmented area were seen in one mouse and a less definite area in the other. Microscopically pigmented cells which were considered to be hepatoma cells were found in both grafts as well as rounded groups of hepatoma cells in the region of the white nodule.

*Sixth week.*—The graft site was easily visible to the naked eye in both mice as a slightly pigmented area. Several small rounded groups of vacuolated and granular hepatoma cells were seen microscopically in one mouse with some pigmented cells adjacent to them. In the second mouse no hepatoma cells were seen.

*Seventh week.*—No graft site could be found in either mouse even after bulk staining.

*Eighth week.*—In one mouse a pigmented area was seen, naked eye, after fixation. Microscopically this was noted to be quite a large area of hepatoma cells extending in finger-like processes into the adjacent fat with a small lymphatic vessel, identified by its content of lymphocytes, at its base. The cells were vacuolated and no mitcses were seen. In the second mouse the site of grafting was not found.

*Ninth week.*—In one mouse a small pigmented area was seen through the dissecting microscope and a few pigmented cells of uncertain nature were found microscopically. The site of grafting was not found in the other mouse.

*Tenth week.*—On naked eye examination a small brown spot was seen at the site of grafting in one mouse. Under the dissecting microscope a small white nodule was noted at the edge of a speckled area. Microscopically this was seen to be composed of a group of hepatoma cells lying in a fatty network. Some of the hepatoma cells were vacuolated and granular and others pigmented. The pigment gave a positive reaction for iron. In the second mouse a similar brown speckled area was found which was composed of actively growing hepatoma cells in which many mitotic figures were seen (Fig. 3).

*Eleventh week.*—Two mice had just palpable grafts with pigmented and liver-like areas on naked eye examination. Microscopically they were actively growing hepatomas in which mitoses were frequent. In one there were small rounded encapsulated groups of hepatoma cells, scattered in fatty tissue around the main tumour, similar to those seen in earlier grafts. Two further mice killed at this time showed a small speckled area at the site of grafting. A few rounded groups of hepatoma cells were seen in one and small clusters of hepatoma cells scattered in a fatty network in the other.

*Twelfth week.*—One mouse had a palpable liver-like graft which was seen to be a trabecular hepatoma showing mitoses. In the second mouse a small pigmented area was seen macroscopically in which, microscopically, there were two small groups of hepatoma cells with flattened cells on the surface encapsulating them. A few scattered hepatoma cells were seen in surrounding fat.

*Thirteenth week.*—Although the grafts were not palpable they were found as small brownish areas and small white nodules were seen through the dissecting microscope in one, in which round nodules of hepatoma with mitoses were found microscopically. In the other a few pigmented cells which were probably hepatoma cells, were seen in a fatty network.

*Fourteenth week.*—In one mouse the graft was identified after fixation and a small rounded group of hepatoma cells and some scattered pigmented cells were found microscopically. In the other mouse a brownish speckled area showed a few pigmented cells which were undoubtedly hepatoma cells.

---

EXPLANATION OF PLATE

FIG. 1.—Vacuolated hepatoma cells lying singly and in a group one week after grafting. ×32[illegible].

FIG. 2.—Encapsulated groups of hepatoma cells lying in fatty matrix four weeks after grating. ×145.

FIG. 3.—Hepatoma graft showing mitoses ten weeks after grating. ×375.

FIG. 4.—Vena cava and liver showing the possible path of metastases from the subcutaneous graft.

1

2

3

4

Head and Laird.

*Fifteenth week.*—In one mouse the graft had been palpable for 2 weeks. It was a solid liver-like tumour with mitoses present. Small rounded groups of hepatoma cells were scattered in the fatty tissue round the main tumour. The second mouse had a tiny graft in which rounded encapsulated groups of hepatoma cells were seen adjacent to other cells which contained round granules of pigment. A third mouse, which was ill, was killed but no hepatoma cells were found although the site of grafting was found histologically.

*Sixteenth week.*—In two mice small pigmented grafts with white areas were seen more clearly after fixation and groups of hepatoma cells with flattened cells encapsulating them were seen histologically. No mitoses were found but some pigment was seen in cells adjacent to the graft. A third mouse which died showed similar features.

*Seventeenth week.*—Macroscopically, small pigmented grafts were found in both mice. After fixation many white nodules were seen in one of them in which groups of hepatoma cells containing mitoses were present. In both grafts, groups of stellate cells lay in a fatty network between the groups of hepatoma cells. Pigmented cells which may be liver cells were also seen.

*Eighteenth, nineteenth, twentieth and twenty-first weeks.*—No definite hepatoma cells were identified in any of the seven mice killed over this period. A small pigmented area was found in three of the mice, and a few pigmented cells were seen in four of them. Only one mouse was killed at the 21st week in order to leave sufficient mice alive.

*Long term findings.*—Three of the remaining five mice then had palpable tumours. They were killed 7 to 8 months after grafting when the tumours were becoming very large and the mice were 10 months old. In one of them, a back cross mouse, two small round yellow tumours were present in the liver, one in the right upper lobe and one in the left half of the middle lobe. They were deeply placed in the centre of the lobes and not standing out from the surface with large blood vessels in grooves as is usual in primary tumours of this type. They were considered to be possible metastases from the hepatoma graft as we have not found primary spontaneous tumours in previous observations in ($C_3$Hf × RIIIf) × RIIIf mice under 14 months old nor have Pullinger and Iversen (1960) seen them in RIIIf mice under 15 months old.

We have traced a possible path of metastases to the liver by injecting white ink into the graft in a freshly killed mouse. The ink can be seen travelling along the iliolumbar vein into the inferior vena cava, thence to the hepatic veins. The liver then becomes white and mottled (Fig. 4) and sections show ink in the hepatic veins within the liver. Multiple metastases to the liver only, from subcutaneous grafts have been found in another experiment in which a lymphoblastoma was being investigated (Head and Chalmers, 1959).

Of the remaining two mice one died after 20 months and one was killed after 21 months. No sign of the graft was found.

TABLE I.—*Number of Mice with Groups of Hepatoma Cells at Site of Grafting*

| | |
|---|---|
| 5–10 weeks | 5/12 |
| 11–21 weeks | 14/25 |
| After 21 weeks | 3/5 |

*Second Series* : 21 *female and* 5 *male mice*

($C_3Hf \times$ RIIIf) $\times$ RIIIf, $F_1$ mice aged 5 to 9 weeks were used. They were grafted subcutaneously by open operation as in the first series and 2 female mice were killed each week for 6 weeks then one at 7 and 8 weeks. The remaining 7 females and the 5 males were kept until a palpable tumour arose or the animal died. This was done in order to obtain information about the length of the latent period in female as well as male mice and to compare the appearances of the graft site in the early stage with the number of tumours in the late stage.

*First week.*—In both mice a small yellow pinhead-sized nodule was found at the site of grafting and a few vacuolated hepatoma cells not forming definite groups were found on microscopical examination.

*Second week.*—A small nodule of graft was found in both mice. Multifocal groups of growing hepatoma cells in which mitoses were present were seen microscopically in one and a few scattered hepatoma cells in the other.

*Third week.*—In both mice a small yellow pinhead-sized graft was found attached to the muscle of the abdominal wall. Groups of growing hepatoma cells, some vacuolated, many binucleated and others in mitoses, were seen in one mouse. In the other a few vacuolated and pigmented hepatoma cells were seen.

*Fourth week.*—In both mice a small yellow graft was found attached to the muscle of the abdominal wall. In addition a smaller nodule in the subcutaneous tissue was found in one. Portions of hepatoma graft showing mitoses were seen microscopically in one mouse. In the other, groups of hepatoma cells were vacuolated and the cells around them contained golden pigment.

*Fifth week.*—In both mice a small yellow graft was seen attached to the muscle of the abdominal wall. In one, groups of vacuolated hepatoma cells were seen lying in a fatty network. In the other, groups of hepatoma cells with a pale granular appearance were surrounded by cells containing golden pigment. No mitoses were seen.

*Sixth week.*—The grafts were seen as small yellow nodules in the subcutaneous tissue and one was also attached to the muscle of the abdominal wall. A few hepatoma cells, some containing golden-coloured pigment and others vacuolated, were found in one mouse and only a few possible liver cells surrounding a necrotic area in the other.

*Seventh week.*—The small graft was attached to the abdominal muscle and microscopically showed groups of hepatoma cells. One group stained well and showed a cell in mitosis and others showed degenerate looking cells. Some cells other than hepatoma cells contained pigment.

*Eighth week.*—A pigmented area smaller than any previous ones in this series was found on the muscle of the abdominal wall. A small group of poorly staining hepatoma cells similar to those seen at seven weeks was found on cutting serial sections of the graft.

At this stage the histological appearance suggested that the graft was degenerating or regressing and the remaining 7 female mice were kept to see in how many the graft had taken. Two months later, 4 months after grafting, tumours became palpable in 3 mice which were killed 7, 10 and 10 months respectively after grafting. All had hepatoma grafts which were very liver-like in appearance and had some cystic and haemorrhagic areas. Two mice were killed in extremis after 19 and 22 months and no graft was found.

The last 2 mice were killed at 26 months. Although no sign of the graft was found in either mouse, a round yellow tumour was present in the centre of the left lobe of the liver in one of them. This was seen histologically to be a hepatoma.

One of the 5 male mice had a palpable graft after 8 weeks and was killed after 7 months when it had a large, pale, fatty-looking hepatoma at the site of grafting. Another male mouse had a palpable graft after 11 months and died after 12 months. This tumour was a golden brown colour. In a third mouse the graft became palpable after 15 months. It was killed after 17 months and had a large brown liver-like graft. Microscopically, all three tumours were typical trabecular hepatomas similar to those in the female mice. Of the remaining two male mice, one died after 24 months and one was killed after 26 months and no sign of the graft was found in either.

TABLE II.—*Number of Mice with Groups of Hepatoma Cells at Site of Grafting*

| | |
|---|---|
| 2–8 weeks . . . | 8/12 |
| After 7 months . . . | 3/7 female |
| | 3/5 male |

*Third Series* : 14 *female and* 5 *male mice*

RIIIf and ($C_3$Hf × RIIIf) × RIIIf 2 to 3-months old mice were used. They were grafted in the same way as the previous series by open operation. In this set it was planned to study the period after 8 weeks when the grafts in the previous series had appeared to regress. However, by the 9th week one female mouse already had a palpable graft and 4 more became palpable by the 10th week. Of the remaining 9 female mice, 2 had tumours at 12 weeks, 1 at 14 weeks, 4 at 19 weeks and 1 at 23 weeks. One female mouse died at 17 months after grafting and the site could not be identified.

Of the 5 male mice, 1 had a palpable tumour at 9 weeks, 3 at 14 weeks and 1 at 19 weeks. Two male mice in this series aged 8 months and 9½ months had small tumours in the liver. The 8-months-old mouse had had a palpable graft for 6 weeks before it was killed and the liver tumour was a small round nodule in the centre of the left lobe. The 9½-months-old mouse had had a palpable tumour for 4 months and the liver tumour was deeply situated, as in metastases, in the right upper lobe, but also projected outwards with large vessels grooving the surface, as in primary tumours. It is debatable whether this is a primary or a secondary tumour.

TABLE III.—*Number of Mice with Palpable Grafts*

| | |
|---|---|
| After 23 weeks . . | 13/14 females |
| | 5/5 males |

*Fourth Series* : 24 *male mice*

Twelve RIIIf and 12 ($C_3$Hf × RIIIf) × RIIIf, $F_1$ 2 to 3 months old mice were grafted subcutaneously from a seventh generation transplant of the hepatoma in order to study further, the period between 5 and 10 weeks after grafting.

In this series 19 of the mice were chosen at random to be killed during this period leaving 5 until a tumour became palpable or the mouse died. This was done in order to compare the number of palpable tumours at the later stage with the histological appearance of the graft sites in the earlier stages (as in the previous series) and to get more information about the variability in the percentage of takes from generation to generation. Twelve of the 19 mice had discrete encapsulated groups of hepatoma cells with mitoses present.

*Fifth week.*—Two mice were killed. In one, groups of hepatoma cells were encapsulated by flattened cells and mitoses were present. In the other no hepatoma cells were found at the site of grafting.

*Sixth week.*—Two mice were killed and in both mitoses were found in groups of hepatoma cells.

*Seventh week.*—Four mice were killed. In 2 of these, groups of hepatoma cells, some showing mitoses, were found at the graft site, which was seen as a group of several small white spots under the dissecting microscope. In a third mouse a few hepatoma cells, not definitely encapsulated, were found. In the fourth mouse no definite liver cells were seen.

*Eighth week.*—Four mice were killed and in 3, small pink nodules were easily found. These were seen, microscopically, to be active hepatoma grafts. In the fourth mouse the site of grafting was not identified.

*Ninth week.*—Four mice were killed and 2 had growing grafts. One of these was a pinhead-sized pink nodule lying in a pad of fat and microscopically showed numerous mitoses. One appeared as a group of small white nodules in the fork of a blood vessel and groups of hepatoma cells were seen near a main lymphatic vessel containing many lymphocytes. In the third mouse a few viable-looking pigmented hepatoma cells were seen lying adjacent to a small lymph node. In the fourth mouse the site of grafting was not found.

*Tenth week.*—Three mice were killed 2 of which had an oval brownish-coloured graft in which mitoses were common. One of these grafts was just palpable. In the third mouse no definite liver cells were found but some macrophages were found containing small homogeneous round granules of pigment.

Of the 5 remaining mice 2 had palpable tumours at 12 weeks, one at 15 weeks and one at 6 months. One died after 24 months with no sign of the graft at the site of implantation.

TABLE IV.—*Number of Mice with Groups of Hepatoma Cells*

| | |
|---|---|
| 5–10 weeks . . . | 12/19 |
| After 12 weeks . . | 4/5 |

### SUMMARY AND CONCLUSIONS

In the first series, 5 of the 12 male mice killed between 5 and 10 weeks after grafting had definite discrete groups of hepatoma cells at the site of transplantation, and 2 showed only a few scattered cells. It was noted that no hepatoma cells were found at the 7th and 9th weeks after grafting. From the 11th to the 21st week 14 of 25 mice had discrete groups of hepatoma cells and 3 only a few scattered hepatoma cells. Mitoses were first seen at the 10th week. In the later period, after 21 weeks 3 of 5 mice had palpable hepatoma grafts.

In the second series 8 of 12 female mice killed between the 2nd and 8th week had discrete groups of hepatoma cells at the site of grafting. In 3 mice only a few scattered hepatoma cells were found. It was noted that at the 8th week the graft appeared less viable than in the earlier weeks. Mitoses were seen in 4 of them at the 2nd, 3rd, 4th and 7th weeks. In the later stage 3 of 7 female mice and 3 of 5 male mice had palpable grafts.

The third series was a continuation of the second series, in order to study the grafts after 8 weeks. The latent period before the earliest graft became palpable was 9 weeks. Thirteen of the 14 female mice and all of the 5 male mice had palpable grafts by the 23rd week. The portions of tissue implanted in this series were larger than in the previous sets and this was considered a possible reason for the higher percentage of takes.

The fourth series was grafted in order to repeat the period from 5 to 10 weeks in male mice. In the early stage from the 5th to the 10th week 12 of 19 mice had definite discrete groups of hepatoma cells and 2 only a few scattered hepatoma cells at the site of grafting. Mitoses were first seen at the 5th week. In the later stage 4 of 5 mice had palpable grafts.

The appearance of mitoses by the 5th week in the fourth series and by the 10th week in the first series indicates that the graft had established itself in a certain proportion of the mice during this time. We originally thought that only the well encapsulated groups of hepatoma cells survived and grew into palpable grafts. However, in view of the number of tumours in the later stage it is probable that some of the scattered hepatoma cells, identified in the early stages, can become active later and may account for the much longer latent period in some mice of the same set.

There are no significant differences between the growth in female and male mice in the generations studied in these experiments.

Some grafts from different tumours in the same generation show a higher percentage of take which may be due to age, or composition of the tumour selected for grafting. In the third series the portions of tissue grafted were larger than in the first two series which may account for more takes in this group.

The latent period, which was taken as the time from grafting to detection of a palpable tumour by weekly examination, varied from 2 to 15 months. In the first generation it was 5 to 14 months but was reduced to 2 to 3 months in the 5th, 6th and 8th generations.

In the later stage of the experiment, mice without palpable tumours were not killed till 20 to 26 months after grafting. One died after 17 months in the third series, and one was killed in extremis in the second series after 19 months.

Possible metastases were found in the liver in 3 mice with large grafts.

Our findings agree with Andervont and Dunn's (1952) that not all grafts grow in early transplant generations although hosts are genetically comparable and the tumours are potentially malignant.

We are grateful to Dr. P. R. Peacock and Dr. B. D. Pullinger for helpful advice and criticism.

### REFERENCES

Agnew, L. R. C. and Gardner, W. U.—(1952) *Cancer Res.*, **12**, 757.
Andervont, H. B.—(1950) *J. nat. Cancer Inst.*, **11**, 581.
*Idem* and Dunn, T. B.—(1952) *Ibid.*, **13**, 455.
Burns, E. L. and Shenken, J. R.—(1940) *Amer. J. Cancer*, **39**, 25.
Head, M. A. and Chalmers, J. G.—(1959) *Rep. Brit. Emp. Cancer Campgn*, **37**, 434.
*Idem* and Laird, H. M.—(1957) *Ibid.*, **35**, 302.—(1958) *Ibid.*, **36**, 405.—(1959) *Ibid.*, **37**, 434.
Laird, H. M.—(1955) *Ibid.*, **33**, 282.
*Idem* and Head, M. A.—(1956) *Ibid.*, **34**, 282.
Pullinger, B. D. and Iversen, S.—(1960) *Brit. J. Cancer*, **14**, 267.


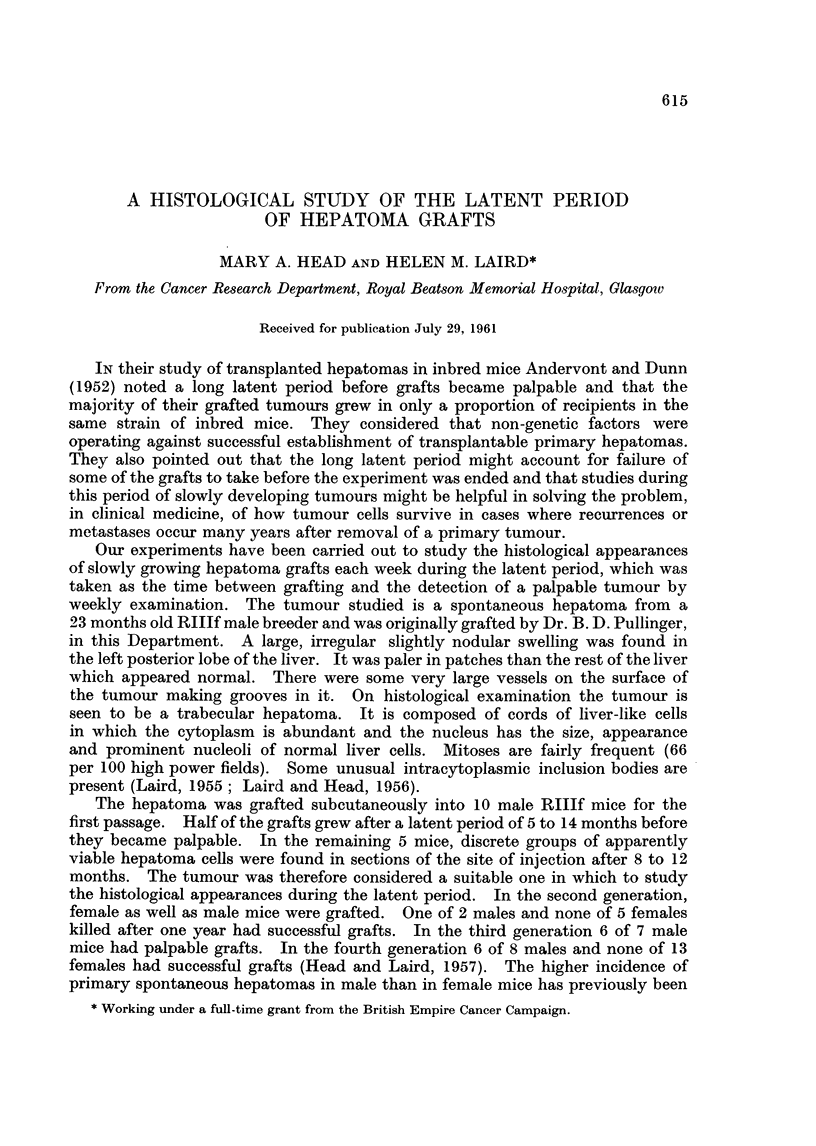

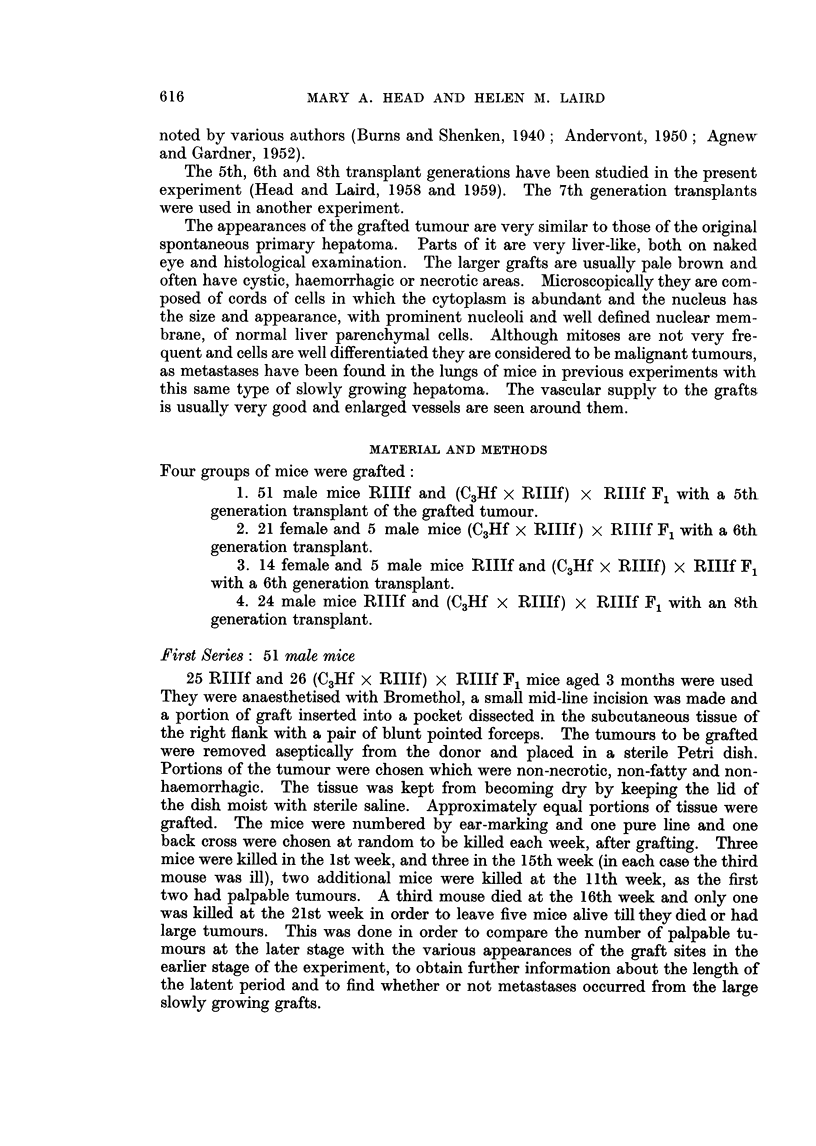

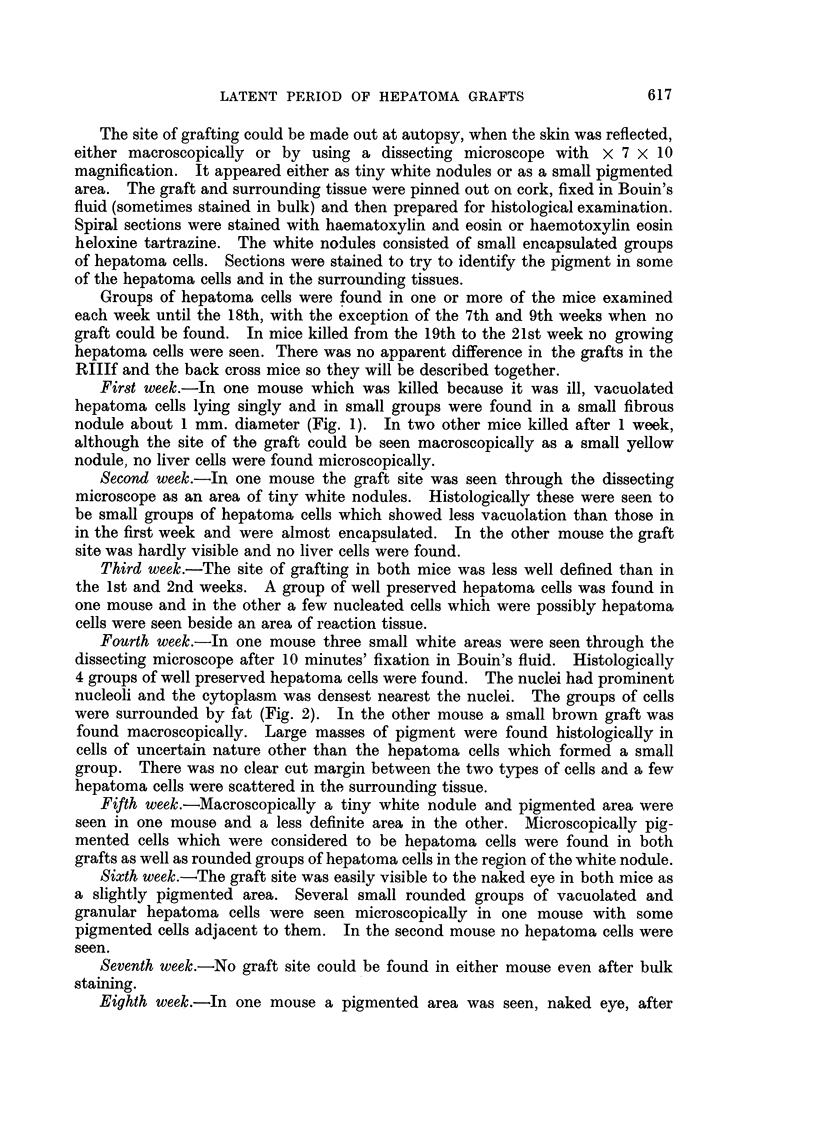

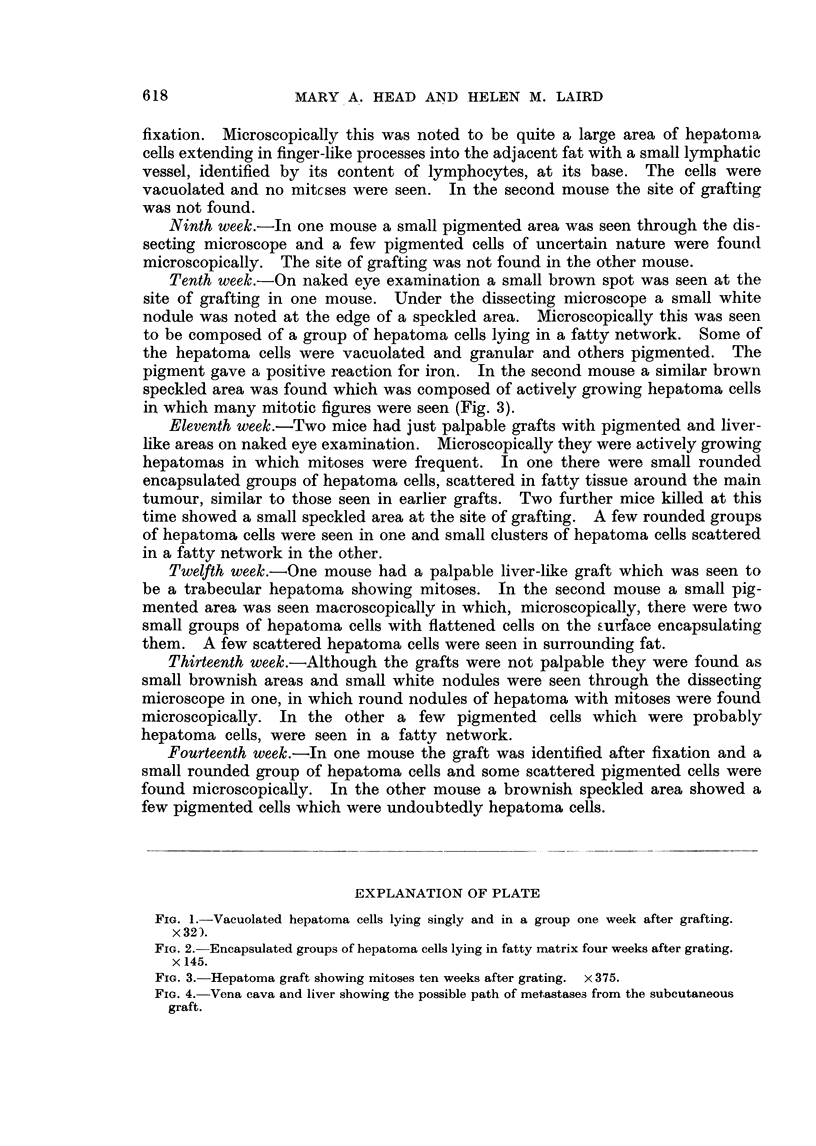

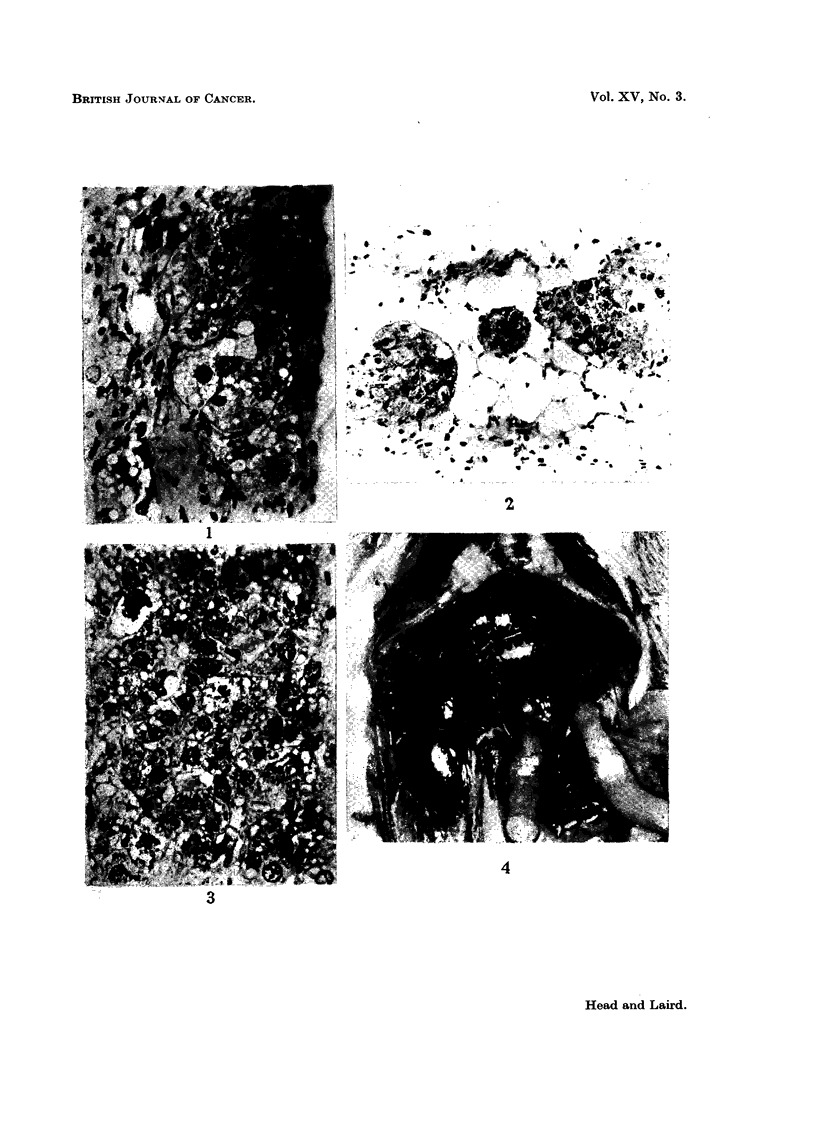

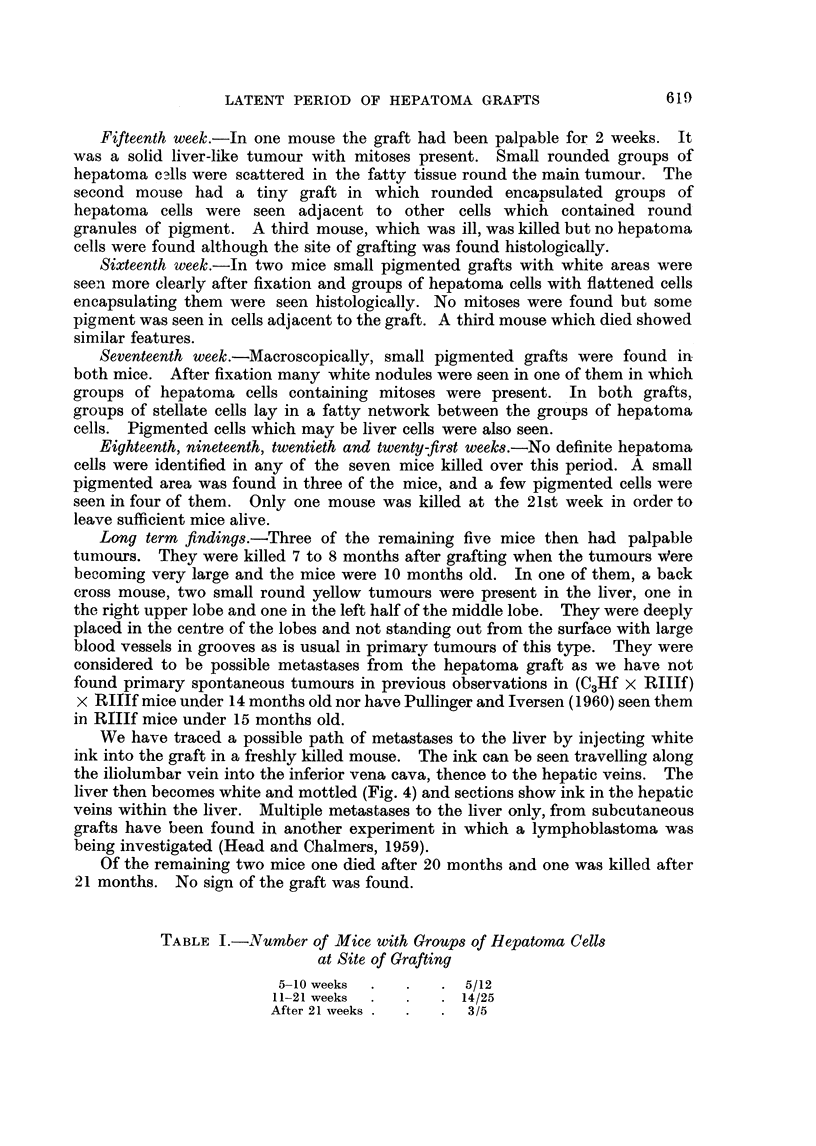

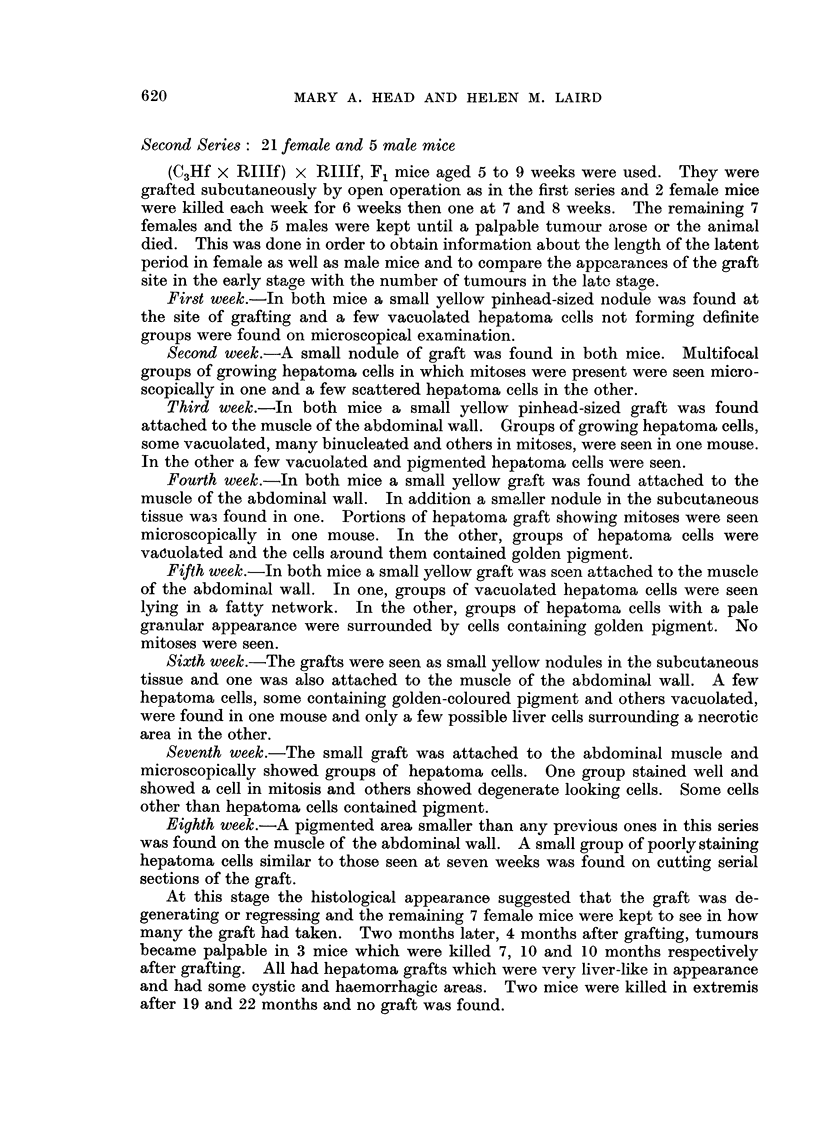

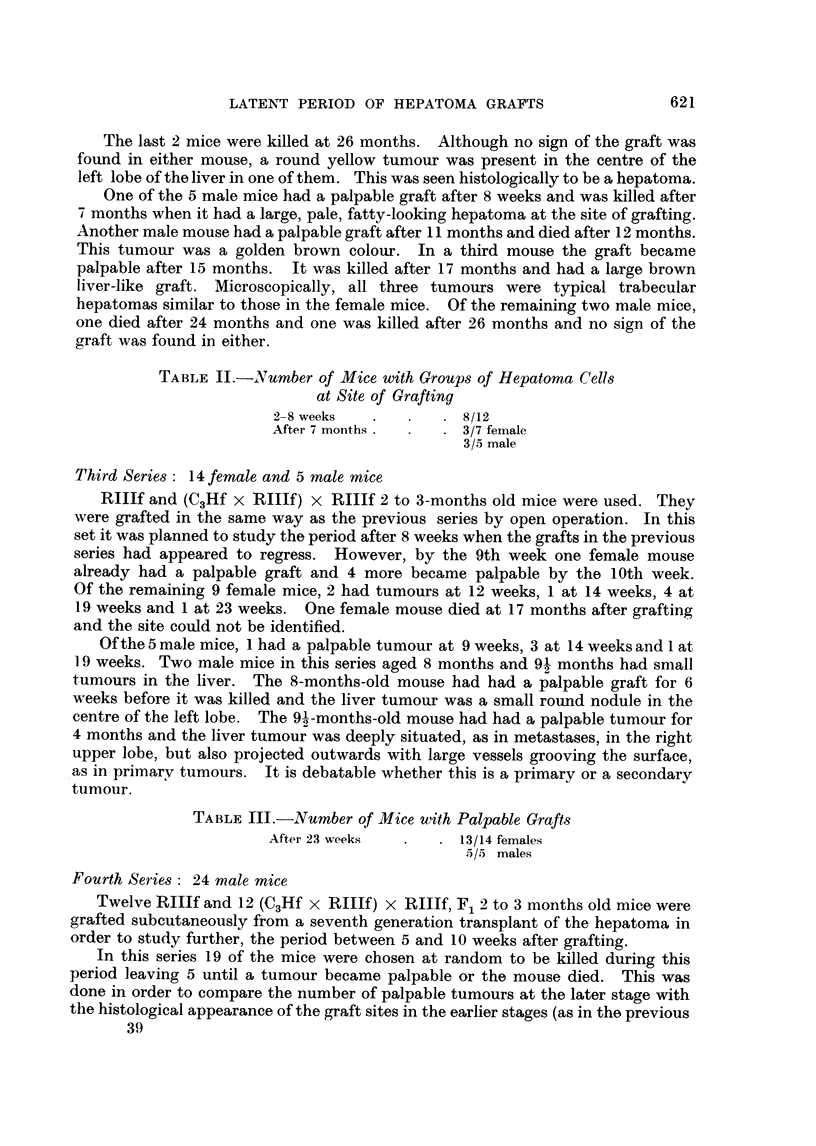

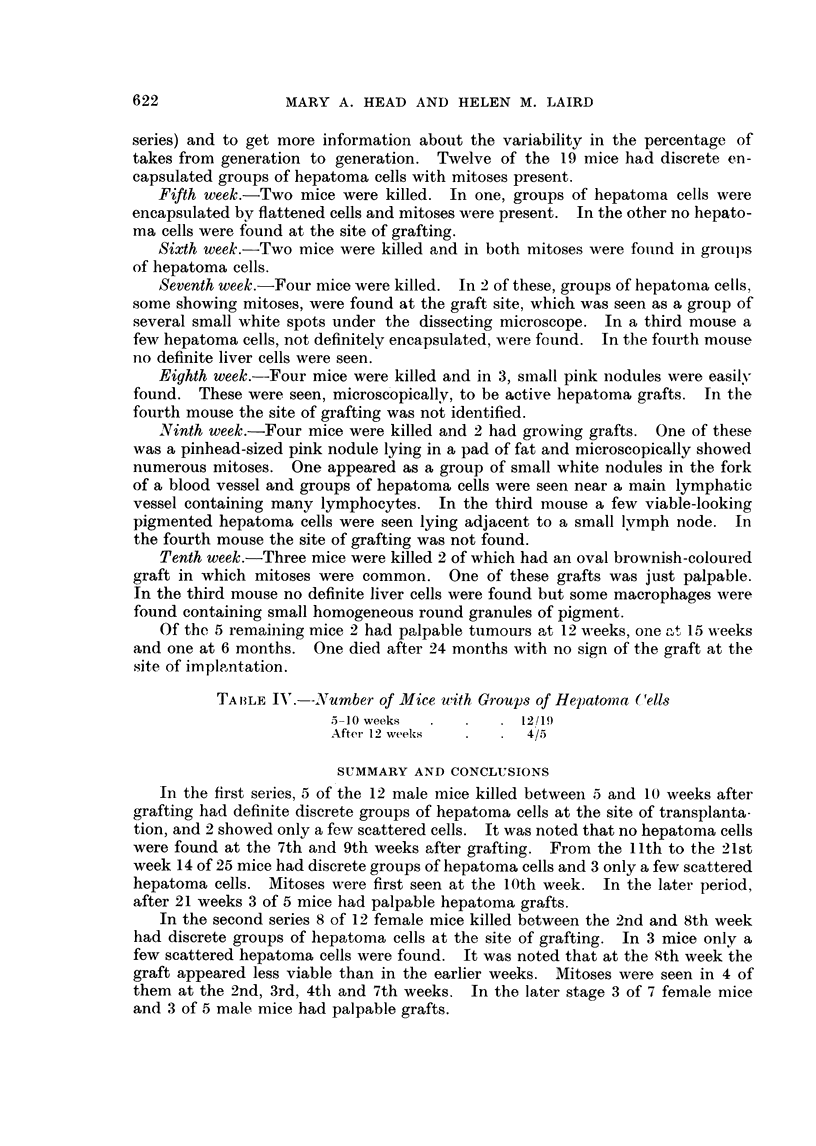

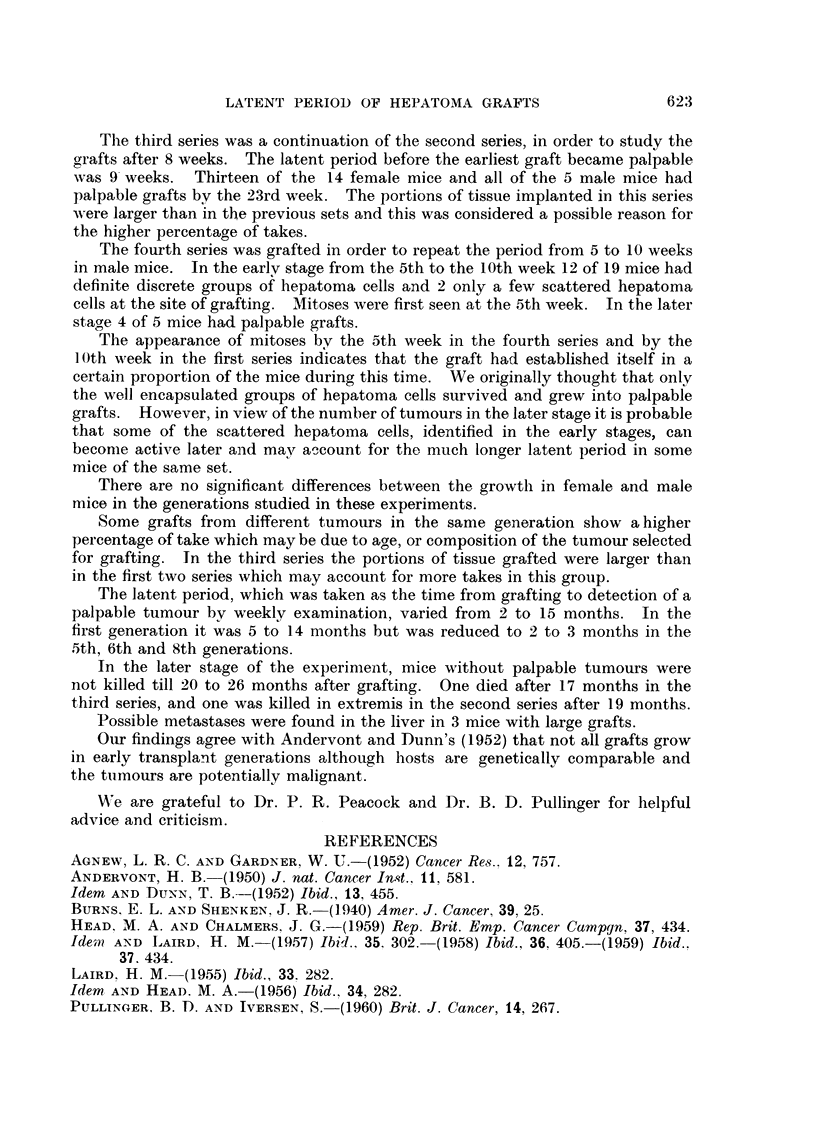


## References

[OCR_00561] ANDERVONT H. B. (1950). Studies on the occurrence of spontaneous hepatomas in mice of strains C3H and CBA.. J Natl Cancer Inst.

